# Design and characterization of UV-driven rechargeable antibacterial nanofibrous membranes for food packaging applications

**DOI:** 10.1016/j.fochx.2025.102665

**Published:** 2025-06-14

**Authors:** Mi Li, Yunge Zhang, Quanlin Li, Weirong Yao, Jian Ju, Yanli Ma

**Affiliations:** a Zhang Zhongjing School of Chinese Medicine, Nanyang Institute of Technology, 80 Yangtze River Avenue, Nanyang, Henan 473004, China; b School of Food Science and Technology, Jiangnan University, 1800 Lihu Avenue, Wuxi, Jiangsu 214122, China; c College of Food Science and Engineering, Qingdao Agricultural University, Qingdao, 266109, China

**Keywords:** Antibacterial packaging, Photodynamic antibacterial, Rechargeable mechanism

## Abstract

This study utilized electrospinning to create in situ composite layers of polyvinyl alcohol (PVA) and polylactic acid (PLA) films. Through molecular grafting, the antimicrobial photoinitiator 3,3′,4,4′-benzophenonetetracarboxylic dianhydride (BD) was anchored to the PVA surface, followed by chlorogenic acid (CA) attachment, forming a composite film with rechargeable photodynamic properties. Scanning electron microscopy showed that the nanofiber structure remained intact post-grafting, while nuclear magnetic resonance confirmed successful bonding of BD and CA with PVA. The composite films exhibited enhanced hydrophobicity, with the PLA/PVA-BDCA film achieving a 44 % increase in contact angle. Antibacterial tests showed significant efficacy in both light and dark conditions, with light exposure doubling the antibacterial rate. The PLA/PVA-BDCA film reduced colony counts by about 3 log units and generated the highest free radical levels (approx. 400 μg/g), over twice that of other films. Ultraviolet irradiation enabled regeneration of its antibacterial function up to five cycles, supporting its sustainable use.

## Highlights

• Successful molecular grafting increased the PLA/PVA-BDCA film's surface contact angle by 44 %.
• PLA/PVA-BDCA film significantly reduced bacterial colony counts, especially under light conditions.
• UV irradiation regenerated the PLA/PVA-BDCA film's antimicrobial performance up to five times.

## Graphical abstract

**Figure:**
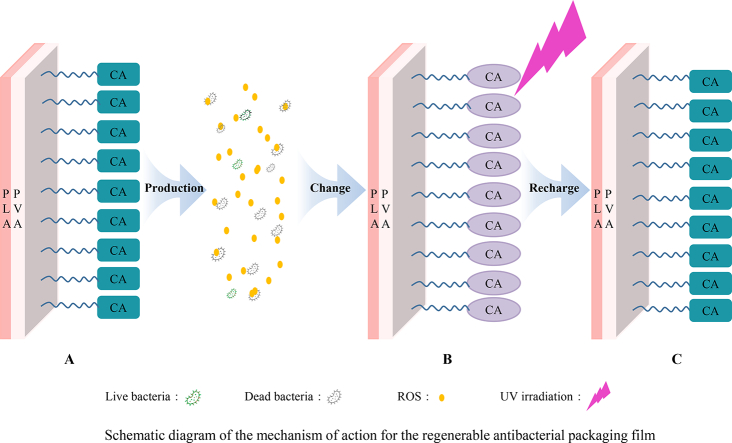

## 1 Introduction

Foodborne pathogens present a substantial threat to public health, the food industry, and socio-economic stability (Zhao et al., 2024). To combat the adverse effects of these pathogens, antimicrobial food packaging films have become a focal point of research. Biopolymers like polylactic acid (PLA), polyvinyl alcohol (PVA), and carboxymethyl cellulose are extensively used in active antimicrobial packaging due to their biodegradability and sustainability (Liu et al., 2024; Rout & Pradhan, 2024; Yang et al., 2024a, Yang et al., 2024b, Yang et al., 2024c). Among them, PLA and PVA stand out as excellent choices for creating eco - friendly active packaging films, given their abundance and renewability (Worku et al., 2024; Yaman et al., 2024). Recent research indicates that electrospun nanofiber composite films made from PLA and PVA outperform single-component films and are conducive to further surface modifications (Andrade et al., 2022; Zhong et al., 2023).

Recently, various antimicrobial agents, such as natural extracts, have been incorporated into biopolymer matrices to endow PLA/PVA-based films with antimicrobial properties (Borges et al., 2024). Nevertheless, the long-term performance of these films is hampered by the potential decline in antimicrobial activity over time and the emergence of bacterial resistance (Gong et al., 2024). In the early stage of food preservation, the relatively low pathogen load allows even weakly antimicrobial packaging films to inhibit bacterial growth. However, as storage time extends, the rapid proliferation of pathogens on the surface of packaged food demands a higher level of antimicrobial activity. Traditional antimicrobial packaging films show strong initial antimicrobial activity, but their efficacy diminishes over time due to the continuous depletion of active substances (Ni et al., 2021). Moreover, some antimicrobial agents may induce bacterial resistance after prolonged use, reducing the long - term antimicrobial efficiency of active substances (Ning et al., 2024). Thus, developing novel packaging films with responsive and durable antimicrobial functions is of utmost importance.

Photodynamic antimicrobial therapy (PAT) is an advanced technology that utilizes photosensitizers and suitable excitation light sources to generate reactive oxygen species (ROS). These ROS can oxidatively damage surrounding biomolecules, including lipids, proteins, and nucleic acids, thereby inhibiting the growth of pathogens (do Prado - Silva et al., 2022; Yu et al., 2022). Unlike other antimicrobial agents, PAT not only effectively inhibits bacteria but also prevents the development of bacterial resistance, as it does not rely on specific targeted interactions with bacteria (Wang et al., 2024a). Combining photosensitizer - doped packaging films with PAT holds great promise for achieving responsive and durable antimicrobial performance (Li et al., 2024a, Li et al., 2024b, Li et al., 2024c; Shi et al., 2022; Wang et al., 2024b). However, if photosensitizers are released from the matrix, the lifespan of these functional materials will be shortened (Chen et al., 2024; Divsalar et al., 2023; Le et al., 2021; Lee et al., 2023; Sudhakar et al., 2024; Yang et al., 2024).

Here, the molecular grafting principle plays a crucial role. In our study, we aim to use this principle to address the issue of photosensitizer release. Molecular grafting is based on the chemical reactions that can occur between the membrane materials and photosensitizers. For example, PVA contains numerous hydroxyl groups in its molecular structure. These hydroxyl groups can react with functional groups on the photosensitizer molecules, such as the anhydride groups in 3,3′,4,4′-benzophenonetetracarboxylic dianhydride (BD). Through esterification or other possible chemical reactions, a covalent bond can be formed between them. Similarly, chlorogenic acid (CA) can also bond with PVA through its hydroxyl - containing functional groups, either directly or after BD is first grafted onto PVA. This covalent bonding ensures that the photosensitizers are firmly attached to the membrane matrix, reducing the risk of release and enhancing the stability and durability of the antimicrobial performance. Chemical grafting of photosensitizers to the matrix can strengthen the bond between them and solve this problem (Zhang et al., 2024a, Zhang et al., 2024b, Zhang et al., 2024c, Zhang et al., 2024d, Zhang et al., 2024e, Zhang et al., 2024f). For example, BD as an organic photosensitizer, can endow PVA-co-PE textiles with excellent antimicrobial properties (Chen et al., 2023a, Chen et al., 2023b). When exposed to sunlight, BD promotes ROS production, showing bactericidal activity and enabling up to 10 cycles of reuse (Si et al., 2018). Despite this, the application of BD-based photosensitizers in designing antimicrobial active packaging films for food remains limited.

Sustained antimicrobial active packaging is crucial for controlling foodborne pathogens and ensuring food safety. In this study, our primary objective was to develop a rechargeable antimicrobial packaging material with broad - spectrum antimicrobial activity and renewable photodynamic properties. We employed electrospinning technology to prepare PLA/PVA composite films and functionalized the surface of PVA nanofibers with different photosensitizer molecules, creating a series of “rechargeable” films with visible-light-driven antimicrobial activity. Specifically, we aimed to: (1) Systematically evaluate the impact of photosensitizer attachment on the morphology, structure, physicochemical properties, and visible-light-driven antimicrobial efficacy of PLA/PVA composite films; (2) Validate the rechargeable antimicrobial performance of the composite films; (3) Elucidate the mechanism of light-driven antimicrobial regeneration. Compared to conventional antimicrobial packaging films, the photodynamic antimicrobial functionality of our composite films can better meet the increasing demand for sustained and effective antimicrobial activity during extended food storage periods. The light-responsive nanocomposite films developed in this study, based on BD and CA, show great potential for advanced food preservation applications in the food industry.

## 2 Materials and methods

### 2.1 Reagents and samples

PLA was purchased from Nature Works LLC (Blair, Nebraska, USA). MgO (50 nm, powder), polyvinyl alcohol (PVA, containing 27 % polyethylene), CA, tetrahydrofuran (THF), carbonyldiimidazole (CDI), polyphosphate (PPA), BD, glutaraldehyde, formaldehyde, potassium iodide, sodium hydroxide, ammonium molybdate tetrahydrate, potassium hydrogen phthalate, *p*-Nitrosodimethylaniline (p-NDA), sodium alginate, deuterated dimethyl sulfoxide (DMSO‑*d*_6_), chloroform, hydrochloric acid, absolute ethanol, acetic acid, dioxane, acetone, and isopropanol were all obtained from Sigma (Nanjing, China).

### 2.2 Preparation of double-layer composite membranes

First, PLA membranes were prepared by tape-casting. To prepare the PLA solution, 2 g of PLA, 0.04 g of MgO, and 50 mL of chloroform were added to a beaker. This solution was then coated onto a clean glass plate to form the PLA membrane. All membranes were stored at 45 °C for 24 h to remove any residual solvent and then kept in a desiccator.

Second, double-layer composite membranes were prepared using electrospinning (LSP02-1B, Baoding Longer Precision Pump Co., Ltd., China). PVA (7.0 g) was dissolved in 100 mL of water by stirring at 85 °C for 4 h to prepare the PVA solution. The previously prepared PLA membranes were attached to the receiver of the electrospinning device to manufacture the double-layer composite membranes using the PVA solution. The electrospinning parameters were as follows: flow rate (2 mL/h), relative humidity (50 %), voltage (28 kV), and collection distance (20 cm). The resulting double-layer composite membranes were designated as PLA/PVA. Finally, the composite membranes were placed between two Teflon layers and compressed at 70 °C for 1 min without applying pressure.

### 2.3 Preparation of rechargeable antibacterial composite membrane

Based on PLA/PVA composite membranes, three new rechargeable antibacterial composite membranes, namely PLA/PVA-BD, PLA/PVA-CA and PLA/PVA-BDCA, were prepared using a modified method from (Si et al., 2018). (1) In the case of PLA/PVA-BD, 1 g of BD and 1 g of PPA were dissolved in 100 mL of dioxane solution. Then, 0.5 g of the PLA/PVA composite membrane was immersed in the prepared solution with stirring at 80 °C. After 2 h of reaction, the resulting membranes were washed with acetone and vacuum dried. (2) In the case of PLA/PVA-CA, 1 g of CA and 25 g of CDI were dissolved in 100 mL THF. Then, 0.5 g PLA/PVA composite membrane was immersed in the prepared solution with stirring at 60 °C. After 2 h of reaction, the resulted membranes were washed with acetone and vacuum dried. (3) In the case of PLA/PVA-BDCA, 1 g of CA and 25 g of CDI were dissolved in 100 mL THF. Then, 0.5 g PLA/PVA-BD composite membrane was immersed into the as-prepared solution with stirring at 60 °C. After 2 h of reaction, the resulted membranes were washed with acetone and vacuum dried.

### 2.4 ^1^H NMR spectrum

The ^1^H NMR spectra of PLA/PVA, PLA/PVA-BD, PLA/PVA-CA and PLA/PVA-BDCA were determined using nuclear magnetic resonance instrument (Avance III 400 MHZ, Bruker, Germany). DMSO‑*d*_6_ was selected as the solvent, and samples were sent after full dissolution.

### 2.5 Reactive oxygen species (ROS)

The primary form of ROS is OH•. The method for determining OH• is as follows: 50 mg of membranes was dissolved in 50 mL of *p*-nitrodimethylaniline solution (p-NDA, 50 μmol/L). The light/dark treatment for this experiment was carried out in a temperature - controlled environment where both the treatment room and the surface temperature were maintained at 25 °C. This temperature was chosen to ensure the stability of the experimental conditions and minimize the influence of temperature on the reaction system, which could potentially affect the generation and detection of ROS.

For the light treatment, the samples were exposed to natural daylight indoors near a window with no direct sunlight blocking. This setup provided a relatively stable light intensity for the reaction. In the dark treatment, the samples were placed in a light-tight cabinet to completely exclude light.

After the solution was prepared, it was exposed to either light or dark conditions for a fixed time interval of 20 min. After the reaction, the absorbance value of the solution was measured at 440 nm using an ultraviolet spectrophotometer. The residual amount of p-NDA in the solution was calculated based on the quantitative curve, and this value was then converted to the amount of OH•.

### 2.6 Water vapor permeability (WVP) and oxygen permeability (OP)

Referred to ASTM E96–95 and adjusted it. All the tests were performed at 25 °C temperature and 90 % RH. First, each composite membranes were cut into a circle having a diameter of 6 cm, and then 15 mL of water was added to each test cup (4.5 cm in diameter and 3 cm in height) to maintain the humidity at 90 %. The membrane was fixed to the mouth of the cup with paraffin. The test cup was placed in a desiccator (relative humidity = 0 %), weighed after 2 h, and then taken out once every 3 h for weighing. WL from each cup was measured as a function of time for 12 h. The test was done in duplicate, and the mean value was reported.

Referred to ASTM D1434–82 (2009) for testing; the experiment used the VAC-V1 oxygen permeability tester (Industrial Physics, Boston, USA). All the tests were performed at 25 °C temperature and 90 % RH. The sample was stored at 25 °C and 50 % RH for 2 h, and then cut into a circle having a diameter of 9.7 cm. The test piece was placed in a tester with a test area of 38.46 cm^2^, and the test time was 8 h at an oxygen pressure of 0.5 MPa.

### 2.7 Contact angle of water and mechanical properties

An OCA 20 AMP contact angle meter (Biolin Scientific AB, Göteborg, Sweden) was used to determine the contact angle of films. Each measurement was repeated ten times.

TA-XT plus texture analyzer (Stable Micro System Ltd., Godalming, UK) were used to test mechanical properties. The A/TG stretching die was selected and calibrated with a 5 kg weight. The initial distance of the holder was set to 50 mm, the test speed was fixed at 1 mm/s, and data were processed using Texture Exponent 32. The tensile strength (TS), modulus of elasticity (EM), and elongation at break (EAB) were calculated by using a stress curve. Each sample was tested eight times, and four parallel samples were taken per sample for a sample size of 10 × 150 mm.

### 2.8 Scanning electron microscopy (SEM)

An SEM FEI Quanta 200FEG (Hillsboro, OR, USA) was used to characterize the surface structure of PLA composite membranes, and the surface was coated with Au/Pd alloy before measurement, using an E5 150 SEM coater (Polaron Equipment Ltd., Doylestown, PA, USA). The pressure was set to 10 kV. The diameters of 30 randomly selected nanofibers from each of the four sample groups were measured using ImageJ software, and the average diameters were determined.

### 2.9 Antimicrobial assay

*E. coli* (ATCC 700728) and *L. innocua* (ATCC 33090) were used as tested bacteria. *E. coli* was inoculated into 10 mL LB agar and cultured at 150 rpm and 37 °C. The absorbance value of the as-prepared solution was measured at 600 nm during the culture process. *L. innocua* was inoculated into 10 mL TSA agar and cultured at 150 rpm and 30 °C. The absorbance value of the as-prepared solution was measured at 600 nm during the culture process. Next, 10 μL bacterial solution was added to the surface of membrane (2 cm × 2 cm), and then the samples were exposed to daylight or dark conditions for a certain time. At each time point, the samples with the bacteria were harvested by vortexing with 1 mL of deionized (DI) water, and the suspension was serially diluted (10^1^, 10^2^, 10^3^, 10^4^, 10^5^, and 10^6^) to be plated on LB agar (*E. coli*) or TSA agar (*L. innocua*) for the bacterial enumeration. Each sample has three parallel sets, and the average number of colonies is recorded.

### 2.10 Protein and nucleic acid leakage

To study the leakage of intracellular proteins and nucleic acids from bacteria treated with the PLA/PVA-BDCA composite film, untreated bacteria were used as the control.

For protein leakage, the absorbance at 260 nm (A260) was measured. After treating bacteria with the composite film, supernatants were collected and analyzed with a spectrophotometer at 260 nm. The A260 differences between treated and control groups evaluated protein leakage. For nucleic acid leakage, the absorbance at 280 nm (A280) was measured. Supernatants from treated bacteria were collected and their absorbance at 280 nm was determined by a spectrophotometer. The A280 differences between groups assessed nucleic acid leakage, helping understand the film's impact on the bacterial cell membrane.

### 2.11 Cucumber collection and packaging

Regarding the fresh cucumbers, they were obtained from a local market in Nanyang, China. To ensure experimental consistency, cucumbers were chosen based on the following specific parameters: length of 12–15 cm and weight of 100–120 g. After collecting, they were quickly transported to the laboratory at a low temperature (maintained around 4 °C during transportation to minimize quality changes) and then divided into groups for packaging experiments. The cucumbers were packaged with antibacterial composite membrane and labeled as PLA/PVA, PLA/PVA-BD, PLA/PVA-CA and PLA/PVA-BDCA, respectively. All samples were stored a 4 °C for 21 days. The following parameters were measured during the storage period (with a 3-day gap). Each sample was repeated three times.

### 2.12 Statistical method

Statistical analyses were performed using one-way analysis of variance (ANOVA) with IBM SPSS Statistics Version 23.0 (IBM Corp., 2015). The least significant difference (LSD) test was applied to identify significant differences between experimental groups, with a significance level set at *p* < 0.05. All statistical results are reported as mean ± standard deviation (SD) to ensure clarity and reproducibility.

## 3 Results and discussion

### 3.1 Characterization of rechargeable antibacterial composite membranes

#### 3.1.1 SEM

**Fig. 1:**
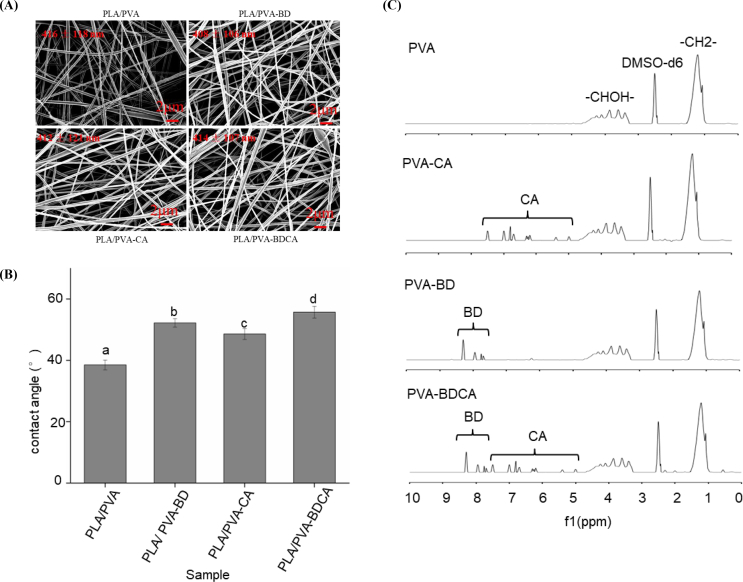
Effect of composite membrane treatment on surface morphology (A); Effect of composite membrane treatment on surface contact angle (the difference of letters between groups indicates that there are significant differences between them, p < 0.05) (B); Effect of composite membrane treatment on ^1^H NMR spectra (C).

Conventionally, rechargeable antibacterial composite membranes were produced by grafting compound molecules on the surface of the membrane. However, this may change the morphology and structure of the membrane. SEM images of the rechargeable antibacterial composite membranes were shown on Fig. 1(A). It is evidenced that the surface of the PVA group exhibits uniform and smooth nanofiber diameters (average 416 ± 118 nm). Upon grafting the molecules BD, CA, and BDCA onto the surface, the microstructure of the nanofibers did not undergo significant changes, which aligns with our anticipated results. Our speculation regarding the main reason was as follows: The molecules (BD, CA, BDCA) that are covalently bonded to the surface of the nanofibers have a comparatively low molecular weight, thus not significantly impacting the morphological structure of the membrane's surface. A similar phenomenon has been observed in the gelatine nanofibers and Polyethylene oxide/Chitosan core-shell nanofibers (Dayisoylu et al., 2023; Zhu et al., 2024). In their study, after modifying the surface of the nanofiber membranes, no significant changes were observed in the smoothness, nanofiber structure, and alignment of the membrane surface. Therefore, surface chemical grafting modification does not alter the surface morphology of the membranes. The primary reason that surface graft modification of membranes does not alter the surface morphology is likely because this modification method operates at the molecular level rather than at the macroscopic structural level. Surface chemical grafting typically involves attaching or introducing functional molecules or groups onto the nanofiber surface. These molecules or groups are very small compared to the diameter of the fibers and the overall membrane structure. Therefore, this modification method does not significantly affect the physical arrangement, structure, and smoothness of the nanofibers.

In another study, Andrade et al. (2022) prepared similar PLA/PVA composite films and characterized their surface structure, with the results as follows: the thickness of different layers ranged from 338 to 374 μm, with 40 % attributed to the central PVA layer (average 160 ± 11 μm) and the remaining 60 % to the external PLA layers (average 101 ± 5 μm). Due to the radial flow of polymer sheets during thermo-compression, the thickness of different layers was reduced, with the PVA layer decreasing by approximately 17 % and the PLA layer by around 10 %. In the P-PVAT and PPVAT-FA multilayer materials, the PVA layer exhibited the most significant thickness reduction (about 20 %) due to the plasticizing effect of glycerol. Furthermore, strong adhesion between the PLA and PVA layers was observed in all multilayer materials, preventing any delamination. The study results validated that the preparation of PLA/PVA bilayer composite films is an ideal choice. Consequently, this research conducted subsequent experiments based on this composite film. In conclusion, the antimicrobial, renewable membrane produced through this research method maintains its surface morphological structure, indicating its potential for application in the field of food packaging.

#### 3.1.2 Contact angle

The contact angle of a membrane is a physical property that describes the interaction between a liquid and the solid surface of the membrane. The contact angle refers to the angle formed by the contact line of a liquid droplet on a solid surface. It can be used to measure the wetting and adhesion properties of the liquid on the solid surface (Sudhakar et al., 2024). The contact angle of water on the surface of the membrane was measured in this study, and the main results are presented in Fig. 1(B). The contact angles of PLA/PVA, PLA/PVA-BD, PLA/PVA-CA, and PLA/PVA-BDCA are 39.25 ± 1.48°, 53.93 ± 2.48°, 51.47 ± 1.80° and 56.10 ± 2.08°. All sample groups exhibit contact angles below 90°, confirming hydrophilic membrane surfaces. Compared to the PLA/PVA control group, the contact angles of PLA/PVA-BD, PLA/PVA-CA, and PLA/PVA-BDCA groups increased significantly (*p* < 0.05). Specifically, the PLA/PVA-BDCA group showed a 42.9 % increase in water contact angle relative to the control(PLA/PVA), suggesting a decrease in hydrophilicity and an enhancement of hydrophobicity after the treatment of the membranes. Drawing on existing research, it has been observed that the incorporation of various natural extracts (curcumin, hydroxyapatite, a tertiary thiophenal quaternary ammonium salt-based antibacterial agent) into nanofiber membranes significantly increased their surface contact angles, similar to our findings (Iqbal et al., 2024; Perikaruppan, Ali, & Shivalingam, 2024; Sudhakar et al., 2024; H. Zhang et al., 2024). The enhanced surface hydrophobicity of membranes after the addition of antibacterial agents is due to several factors. Antibacterial agents are often inherently hydrophobic and form a hydrophobic layer on the membrane surface. They interact with hydrophilic groups in the membrane, reducing surface energy and increasing hydrophobicity. Additionally, they can alter the membrane's microstructure, increasing surface roughness, which traps air and reduces liquid-solid contact. Some agents introduce hydrophobic groups through chemical reactions, and others form a physical barrier, preventing water contact. These combined effects enhance the membrane's water repellency and anti-fouling properties. Consequently, the introduction of active functional components into nanofiber membranes generally enhanced the hydrophobicity of the membrane surface.

#### 3.1.3 ^1^H NMR spectrum

In the quest to determine whether BD, CA, and BDCA molecules were covalently bonded to the surface of polyvinyl alcohol molecules, this study employed ^1^H NMR spectroscopy on four distinct types of membranes, as vividly illustrated in Fig. 1(C). A meticulous examination reveals that the NMR spectrum of the PVA membrane displays only three characteristic peaks. Through careful comparison, it can be discerned that, from left to right, these peaks correspond to -CHOH-, the DMSO‑*d*_6_ reference, and -CH_2_-. This is in precise agreement with the well-known molecular structure model of polyvinyl alcohol, providing a basis for our subsequent analysis.

When delving deeper into the spectra of the other three membranes, in addition to the aforementioned three peaks, distinct additional peaks corresponding to CA and BD become apparent. What's more, the positions of the peaks representing identical structures in these three membranes exhibit remarkable similarity. This consistency in peak positions not only validates the reliability of our experimental data but also provides strong evidence for the successful covalent attachment of CA and BD to the PVA matrix.

As reported in a study by Smith et al. (2024), in a similar polymer-photosensitizer system, the appearance of new peaks and the retention of original polymer-related peaks in the NMR spectrum were considered reliable indicators of covalent bonding. In our case, considering all four spectra comprehensively, we can firmly confirm that CA, BD, and BDCA molecules have indeed formed covalent linkages with the polyvinyl alcohol molecules. This covalent bonding not only serves as the cornerstone for their antibacterial activity but also endows the composite membrane with the remarkable ability to regenerate its antibacterial properties, a crucial aspect for sustainable food packaging applications.

Based on the chemical structures of PVA, BD, and CA, we propose the following bonding mechanisms: The surface of PVA molecular chains is rich in hydroxyl groups (-OH), while the benzene ring structure of BD contains carboxyl groups (-COOH). CA molecules possess both hydroxyl (-OH) and carboxyl (-COOH) functional groups. In the PLA/PVA-BD and PLA/PVA-CA systems, the hydroxyl groups on the PVA surface can undergo esterification reactions with the carboxyl groups of BD and CA molecules, respectively, forming ester bonds and achieving covalent attachment. In the case of the PLA/PVA-BDCA system, BD first anchors onto the PVA surface through the esterification reaction. Subsequently, the carboxyl group of BD further reacts with the hydroxyl group of CA via esterification, constructing a “PVA-BD-CA” covalently linked chain structure.

Furthermore, a wealth of existing literature, such as the work by K. Zhang et al. (2024), has demonstrated that covalent bonding of molecules to the membrane surface offers unparalleled stability compared to other attachment methods. This type of bonding effectively minimizes the loss of migrated molecules, a key advantage for active packaging membranes. It ensures that the antibacterial agents remain firmly attached to the membrane surface, even under challenging conditions, thereby enhancing the overall performance and longevity of the packaging material.

When it comes to antibacterial membranes, surface chemical bonding of antibacterial agents presents a plethora of advantages over direct addition. Antibacterial agents chemically bonded to the membrane surface form robust covalent bonds, providing an extremely stable attachment. This stable connection prevents the antibacterial agents from being washed away or migrating during the practical use of the packaging material, thereby ensuring the long-term stability of the antibacterial effect (P. Harini et al., 2025). In contrast, directly added antibacterial agents often suffer from a gradual decline in efficacy over time and with repeated use. Chemically bonded antibacterial agents, on the other hand, enable a sustained release of antibacterial activity, offering continuous and long-lasting protection against harmful pathogens.

Chemical bonding also plays a crucial role in reducing the wastage of antibacterial agents. By minimizing migration and loss, it significantly improves the utilization efficiency of the antibacterial agents, ultimately extending the lifespan of the antibacterial membrane. Moreover, this bonding mechanism allows for a more uniform distribution of antibacterial agents across the membrane surface. As a result, the membrane exhibits consistent antibacterial performance throughout its entire structure, effectively avoiding the uneven distribution issues commonly associated with direct addition methods.

In summary, surface chemical bonding of antibacterial agents is far superior to direct addition. The numerous benefits of this approach stem primarily from the stability and uniformity conferred by chemical bonds. These bonds not only guarantee the durability and safety of the antibacterial effect but also contribute to enhancing the overall performance of the antibacterial membrane, making it an ideal choice for advanced food packaging applications.

### 3.2 Barrier properties of the rechargeable antibacterial composite membranes

**Fig. 2:**
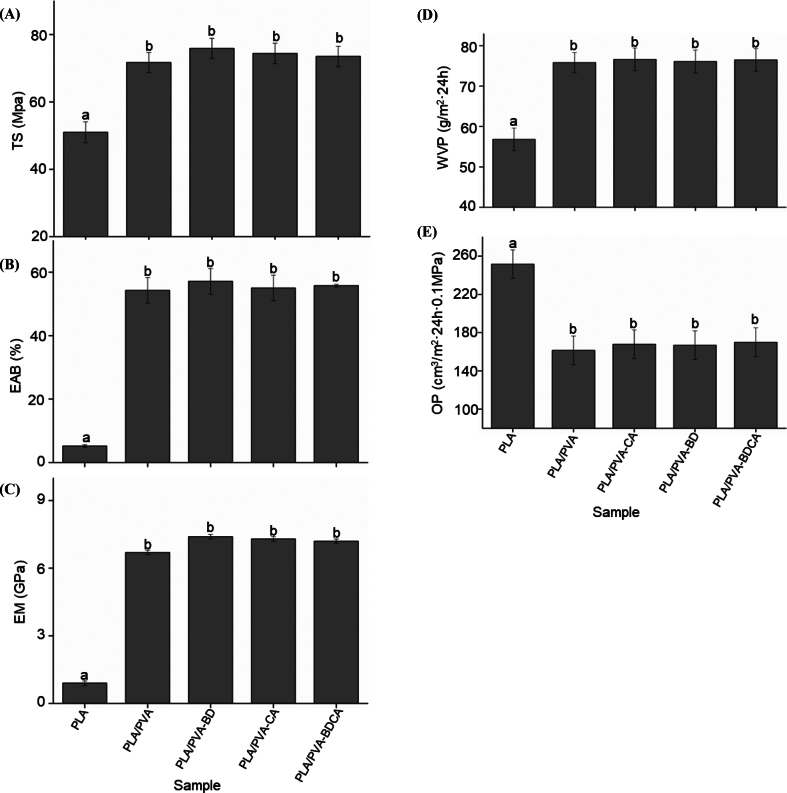
Effect of composite membrane treatment on mechanical properties: TS (A), EAB (B), EM (C)); Effect of composite membrane treatment on barrier performance: WVP (D), OP (E).

In the context of food packaging, the permeation of small molecules from the environment, such as water vapor and oxygen, into the internal environment can lead to food oxidation and extensive microbial proliferation, resulting in food spoilage (S. Chen et al., 2023). Consequently, the barrier properties of food packaging films are crucial for their practical application value, as inadequate barrier performance can fail to meet the requirements of effective food preservation. During the preparation of the composite membrane, MgO nanoparticles were incorporated into the PLA-based matrix to enhance the barrier properties of the composite membrane (Yang et al., 2024). In this study, the oxygen transmission rate (OTR) was measured to evaluate the material's ability to limit oxygen ingress, as excessive oxygen can accelerate oxidative reactions and promote the growth of aerobic spoilage microorganisms. This assessment is particularly relevant to the packaging of fresh produce, such as cucumbers, where controlling oxygen levels inside the package can significantly impact shelf life. Therefore, in this section, the barrier properties of the rechargeable antibacterial composite membranes were assessed, with the results depicted in Fig. 2(D, E).

As shown in Fig. 2(D, E), there were no significant differences in the oxygen permeability and water vapor permeability among the three composite films (PLA/PVA-BD, PLA/PVA-CA, and PLA/PVA-BDCA) compared to the PLA/PVA composite film (p ˃ 0.05), indicating that the surface grafting treatment did not alter the barrier properties of the composite films. The study by Andrade et al. (2022) also showed that the barrier properties of PLA/PVA composite films did not significantly change after surface modification, but there was a significant increase in barrier properties compared to single-layer films. In another study, Cheng et al. (2024) prepared CS/PVA nanofiber composite membranes. After directly adding the antibacterial agent 4-terpineol/β-cyclodextrin inclusion complexes, the barrier properties of the composite membranes were significantly enhanced. This differs from our experimental results. The likely reason is that the addition of the antibacterial agent altered the physical and chemical properties of the membrane, such as its density, crystallinity, and molecular chain arrangement. These properties are key factors in determining the barrier performance of the membrane, leading to an improvement in its barrier properties.

The surface graft modification of membranes does not alter their barrier properties primarily because this modification method operates at the molecular level rather than at the macroscopic structural level. The reasons are as follows: surface graft modification involves molecular or atomic-level operations on the membrane surface. These modification molecules are relatively small, and the modification process does not significantly change the overall structure or thickness of the membrane. Therefore, the macroscopic structure and overall density of the membrane remain unchanged, which does not significantly affect its barrier properties. Additionally, the chemical modification typically forms an extremely thin modification layer, much thinner than the total thickness of the membrane. Since barrier properties are mainly determined by the overall thickness and density of the membrane, a thin surface modification layer does not significantly impact the barrier performance of the membrane.

In comparison with the PLA composite film, the water vapor transmission rate significantly increased, and the oxygen transmission rate significantly decreased for the four films after composite treatment (*p* < 0.05), suggesting a significant enhancement in the barrier properties of the four films after composite treatment. This enhancement is attributed to the increased thickness of the films and the fact that PVA is a material known for its excellent barrier properties. Existing literature indicates that PVA films, owing to their excellent barrier properties, are suitable for food packaging applications with high barrier requirements (Y. Liu et al., 2023). They have demonstrated favorable barrier properties when used alone to prepare antimicrobial or antioxidant packaging, or when mixed with other materials (Cheng et al., 2024; B. Li et al., 2024).

### 3.3 Mechanical properties of the rechargeable antibacterial composite membranes

The mechanical properties of packaging films are integral to their effectiveness in protecting and preserving products, enhancing user experience, and contributing to economic and environmental efficiency. Ensuring that packaging films possess optimal mechanical properties is essential for meeting the diverse demands of modern packaging applications. Therefore, during the preparation of the composite membrane, MgO nanoparticles were added to the PLA-based matrix to improve the mechanical properties of the PLA/PVA composite membrane (Yang et al., 2024). In this section, we measured the TS, EAB, and EM of the PLA/PVA composite membranes.

As shown in Fig. 2(A, B, C), there are no significant differences in the mechanical properties of the composite membranes obtained through the three treatments (PLA/PVA-BD, PLA/PVA-CA, and PLA/PVA-BDCA) compared to the PLA/PVA composite membrane (p ˃ 0.05), indicating that surface grafting treatment does not alter the mechanical properties of the composite membranes. According to Yong's et al. (2024) study, the macroscopic mechanical properties of the composite films showed significant improvements in tensile strength and elongation at break, particularly for the SS-ε-PL-TA composite film, which achieved a tensile strength of 64.34 ± 6.22 MPa and an elongation at break of 13.98 ± 0.22 %. This enhancement is primarily attributed to enhanced cross-linking and chain entanglement facilitated by the interactions between TA and ε-PL. This differs from our results, where the main reason could be that surface graft modification primarily enhances the surface functionalities of the membrane, such as antibacterial and antifouling properties, rather than modifying the base structure of the membrane. Therefore, the modification mainly improves the surface performance without adversely affecting the mechanical properties.

Compared to the base membrane (PLA), the elongation at break, tensile strength, and elastic modulus of the four composite-treated membranes are significantly increased (*p* < 0.05), indicating a substantial improvement in mechanical properties after composite treatment, especially the elongation at break, which is ten times that of the base membrane. This enhancement is partly due to the increased thickness of the membrane and partly because PVA is a material with excellent mechanical properties. Existing literature indicates that PVA membranes exhibit outstanding EAB, effectively compensating for the poor toughness of the base membrane. Whether used alone for the preparation of antibacterial or antioxidant packaging or in combination with other materials, PVA demonstrates excellent mechanical properties (Cheng et al., 2024; B. Li et al., 2024).

### 3.4 Antimicrobial activity

The rationale for testing *E. coli* and L. *monocytogenes* lies in their high pathogenicity and frequent association with foodborne illnesses. These microorganisms are widely recognized as model organisms in food safety research due to their ability to contaminate various foods, posing significant risks to public health and highlighting the importance of developing effective antimicrobial packaging solutions.

**Fig. 3:**
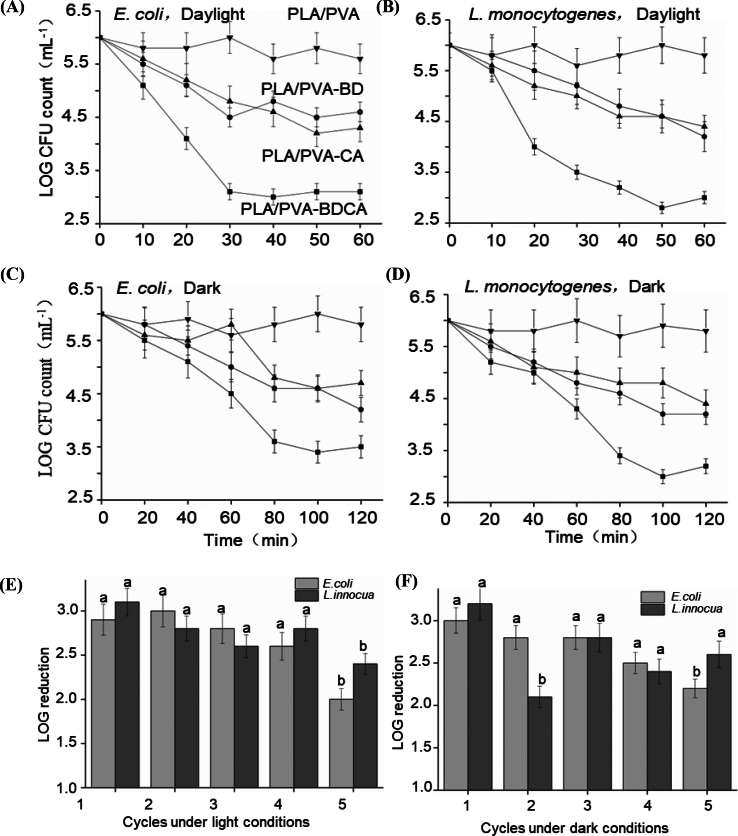
Bacteriostatic effect of four membranes on *E. coli* and L. *monocytogenes* under light (A and B) and dark (C and D); Bacteriostatic effect of PLA/PVA-BDCA membrane reused for five times (E-light, F-dark) (the different lowercase letters indicates significant differences in the group, *p* < 0.05).

Fig. 3 presents the results of the antimicrobial tests conducted on the composite films using Gram-negative bacteria *E. coli* and Gram-positive bacteria L. *monocytogenes*. Initially, the antimicrobial performance of the composite films under light conditions was tested, as shown in Fig. 3(A, B). After 60 min of treatment with PLA/PVA composite films, there was no significant reduction in the total colony count, with the final logarithmic values of colony counts being approximately 5.6 and 5.8, respectively. This indicates that the control group samples did not exhibit any antibacterial effects against these two bacteria. In contrast, the PLA/PVA-BD and PLA/PVA-CA samples both showed a reduction of approximately 1.5 in the logarithmic values of the colony counts for *E. coli* and L. *monocytogenes*, indicating a certain level of antibacterial effectiveness for both, with similar effects on the two types of bacteria. Compared to the previous groups, the PLA/PVA-BDCA samples demonstrated the best antibacterial performance against both *E. coli* and *L. monocytogenes*, with a reduction of approximately 3 in the logarithmic values of colony counts within 60 min, and a significantly higher antibacterial rate than PLA/PVA-BD and PLA/PVA-CA (*p* < 0.05).

Based on the experimental phenomena described above, it can be concluded that the composite membrane with surface grafted BD, CA, or BDCA molecules exhibits good antibacterial effects against both Gram-positive and Gram-negative bacteria, with BDCA showing the best antibacterial effect, superior to its individual use. This may be due to the following reasons: the antibacterial effect of the composite membrane depends on the oxygen radicals produced by the photosensitizer, which are non-selective in their antibacterial activity; additionally, the bonding of the photosensitizer with chlorogenic acid enhances the ability to generate oxygen radicals, thereby strengthening the antibacterial effect (Du et al., 2024). Comparative studies have shown that antibacterial packaging films prepared by various researchers also achieved good antibacterial effects using antibacterial materials such as various plant essential oils and silver ions (H. Guo et al., 2024; Ibeogu et al., 2024). Chen et al. (2024) prepared composite films with photodynamic antibacterial activity using a photosensitizer (curcumin) and zinc ions. They found that both curcumin and zinc ions alone exhibited photodynamic antibacterial effects. However, the combined use of Cur-Zn demonstrated significantly stronger antibacterial activity against various food spoilage bacterial strains. At a concentration of 1 mg/mL, Cur-Zn reduced the colony-forming units (CFU) of *Pseudomonas aeruginosa*, *E. coli O157*, *Proteus mirabilis*, and *non-toxigenic E. coli* (ATCC 25922) by 1.3, 1.3, 1.0, and 1.7 log CFU/mL, respectively. In contrast, treatment with curcumin alone reduced the CFU of these microorganisms by only 0.2–0.4 log CFU/mL. The enhanced antibacterial effect of Cur-Zn is primarily attributed to its inherent and PDI-enhanced activities, rather than the effect of Zn(OAc)^2^. These findings are consistent with our experimental results.

Subsequently, the antibacterial performance of the composite films under dark conditions was tested, as shown in Fig. 3(C, D). In the control group, the total colony counts of the two bacteria did not significantly decrease within 120 min, with final logarithmic values of approximately 5.8 and 6.0, respectively, indicating no antibacterial effect of the control group samples under dark conditions. In contrast, the PLA/PVA-BD and PLA/PVA-CA samples both showed a reduction of approximately 1.5 in the logarithmic values of colony counts for *E. coli* and L. *monocytogenes*, indicating a certain level of antibacterial effectiveness under dark conditions, with similar effects on the two types of bacteria. Compared to the previous groups, the PLA/PVA-BDCA samples also exhibited the best antibacterial performance under dark conditions against both *E. coli* and *L. monocytogenes*, with a significantly higher antibacterial rate than the other two groups (*p* < 0.05). Within 120 min, the logarithmic values of colony counts decreased by approximately 2.5 and 3, respectively. These results indicate that the antibacterial films exhibit similar antibacterial effects against Gram-positive bacteria L. *monocytogenes* and Gram-negative bacteria *E. coli*, and that light is not a necessary condition for the antibacterial effect. However, achieving the same antibacterial effect under dark conditions takes twice as long as under light conditions, with the antibacterial rate being faster under light conditions (Si et al., 2018). Comparative studies have shown that researchers using similar antibacterial materials have achieved better antibacterial effects, with total colony counts reduced to zero within 24 h (Cheng et al., 2024). This suggests that the antibacterial performance of the membrane materials prepared in this study can be further improved, possibly due to the suboptimal effect of the grafting treatment and the limited number of effective functional groups generated, hindering the optimal antibacterial effect (Gong et al., 2024). Considering the antibacterial effects of the four composite films under both light and dark conditions, we found that the composite film with BDCA molecules grafted onto the surface exhibited the best antibacterial performance. Therefore, for subsequent antibacterial regeneration experiments, we selected the PLA/PVA-BDCA composite film as the subject of study.

### 3.5 Characterization of antimicrobial renewable effects

This section tested the reuse cycles of the antimicrobial composite film, with the results illustrated in Fig. 3(E, F). The results show that after five cycles, the composite film became damaged and lost its usability. During the reuse process, the antimicrobial effectiveness of the composite film exhibited a decreasing trend, likely due to the destruction of the film's surface structure and the reduction of antimicrobial groups during use. However, the antimicrobial effect persisted throughout the five cycles until the film was damaged.

To better understand the significance of our findings, it is crucial to compare them with previous research. González-Ceballos et al. (2022) developed a reusable antimicrobial film aimed at food preservation. Their film relied on the addition of specific chemical reagents to rejuvenate its antimicrobial properties. These chemical reagents were complex to handle and required precise dosing to ensure the film's renewed efficacy. For instance, they had to carefully control the concentration of an antibacterial agent solution during the regeneration process. This not only added an extra step in the film's application but also raised concerns about potential chemical residues in food products, which could pose risks to consumer health.

Li et al. (2021) also explored the development of reusable antimicrobial films. Their approach involved incorporating metal-organic frameworks (MOFs) into the film matrix. Although their film showed good initial antimicrobial performance, the regeneration process was equally challenging. To regain its antibacterial activity, the film needed to be immersed in a solution containing a specific ligand that could reactivate the MOFs. This process was time-consuming and required specific environmental conditions, such as a narrow pH range and a controlled temperature.

Yang et al. (2023) designed a composite film with antibacterial properties. Similar to the above-mentioned studies, their film's regeneration relied on chemical treatments. They used a chemical bath to re-introduce antibacterial substances into the film structure. However, this method led to inconsistent results in terms of the film's long-term stability and antimicrobial efficacy. The repeated chemical treatments gradually degraded the film's physical properties, reducing its mechanical strength and flexibility.

Zhang et al. (2022) developed a reusable antimicrobial film for wound dressing applications. Their film required a chemical - induced cross - linking reaction to restore its antibacterial effect. This process was complex and often led to changes in the film's surface texture, which could potentially affect its biocompatibility when used in contact with human tissues.

In contrast, the PVA-BDCA composite film developed in this study can be reused up to five times without the need for additional chemical reagents. Whether under light or dark conditions, our film's antibacterial performance is self-renewable to a certain extent. This is mainly attributed to the unique photodynamic mechanism of the BDCA moiety. When exposed to light, BDCA can generate ROS that can continuously kill bacteria, thus maintaining the film's antimicrobial effect. This not only simplifies the application process but also eliminates concerns about chemical residues and complex regeneration procedures. Therefore, the composite film developed in this study presents a more advantageous solution, offering a more sustainable and user-friendly option for applications in food packaging and potentially other fields where antimicrobial films are required.

### 3.6 Investigation into the renewable mechanism of antimicrobial effects

#### 3.6.1 The generation of ROS

**Fig. 4:**
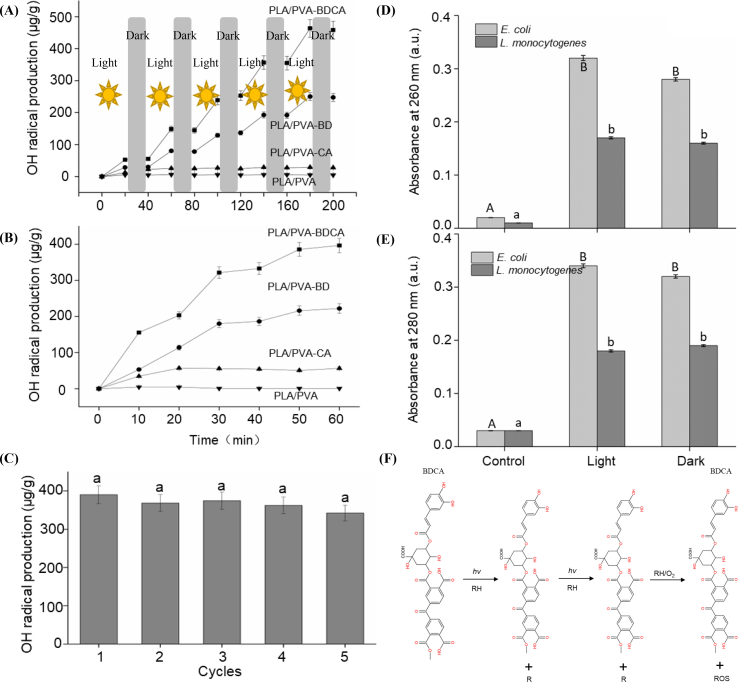
Variation of OH radical generation with time in four films under light/dark conditions with an interval of 20 min (A); Changes of OH radical production with time under dark conditions after illumination for one hour (B); Amount of OH radical generated from PLA/PVA-BDCA membrane is recycled (C); Leakage of protein (D) and nucleic acid (E) of *E. coli* and *L. monocytogenes* after PLA/PVA-BDCA membrane treatment (control group: two kinds of bacteria without composite membrane; the different lowercase letters indicates that there are significant differences in the same bacteria treatment group, p < 0.05); Schematic diagram of ROS recycling in membrane (F).

The generation of free radicals by the composite film was analyzed. Results shown in Fig. 4(A) illustrate the production of free radicals under intermittent UV light exposure (with intervals of 20 min), representing the process of charging the antimicrobial film to activate its antimicrobial properties post-fabrication. Fig. 4(B) depicts the generation of free radicals within 60 min after the antimicrobial film has been fully exposed to UV light (completion of the charging process), simulating the antimicrobial action of the film.

Analyzing Fig. 4(A), it is evident that the composite film treated solely with PLA/PVA does not generate free radicals under intermittent UV light, indicating no antimicrobial effect and inability to acquire antimicrobial properties through UV irradiation. The PLA/PVA-CA composite film produced approximately 30 μg/g of free radicals within the initial 20 min of exposure, with no further increase during subsequent irradiation. This suggests a limited capacity for free radical generation, as it becomes unaffected by further UV light once a certain concentration is reached. In contrast, the composite films treated with PLA/PVA-BD and PLA/PVA-BDCA continuously generated free radicals with UV light charging, with stable free radical concentrations during non - irradiation periods, and increased generation upon re - exposure to UV light. This indicates that UV light is essential for regenerating the antimicrobial capacity of the composite film (S. Chen et al., 2023). However, it should be noted that the antibacterial performance of the film is significantly influenced by external conditions such as light intensity and temperature. Under low-light conditions, the generation of ROS by the photosensitizers may be substantially reduced, thereby weakening the antibacterial effect. Similarly, extreme temperatures can impact the stability of the film structure and the activity of the antibacterial groups, potentially leading to a decline in the overall antibacterial performance. Compared with other literature, we found that ultraviolet or visible light is a necessary condition for producing photodynamic antibacterial activity (Chen et al., 2024; de Moraes Pinto et al., 2023; Divsalar et al., 2023; Gong et al., 2024). The antibacterial performance of the antibacterial film will be enhanced or reactivated after exposure to light (Le et al., 2021; L. Li et al., 2024). This significantly enhances the practicality of food antibacterial films.

From Fig. 4(B), the composite film treated only with PLA/PVA did not generate free radicals within 60 min, thus lacking antimicrobial properties. The PLA/PVA-CA composite film produced approximately 30 μg/g of free radicals in the initial 20 min, with no further change in free radical content, explaining its antimicrobial effect under dark conditions due to the presence of a small number of free radicals. The composite films treated with PLA/PVA-BD and PLA/PVA-BDCA continuously generated free radicals under dark conditions until the concentration stabilized, with the number of free radicals generated by PLA/PVA-BDCA being approximately twice that of PLA/PVA-BD. This explains why the PLA/PVA-BDCA composite film exhibits the best antimicrobial effect under dark conditions, a conclusion also reached by other researchers (S. Chen et al., 2023; Si et al.). These results indicate that the PLA/PVA-BDCA composite film prepared in this study can continuously generate free radicals under dark conditions, akin to a battery discharging after being fully charged. Compared with traditional photodynamic antibacterial technology, the antibacterial film prepared by this method still has good antibacterial effect during non - light periods (L. Li et al., 2024; Y. Liu et al., 2023; Shi et al., 2022). This significantly expands the application scope of the antibacterial packaging. For example, intermittent light exposure can maintain good antibacterial effects on products during storage, with significant advantages compared to continuous light exposure. However, reliance on specific environmental conditions, such as appropriate light intensity and temperature, remains a factor that could limit the consistent effectiveness of the film in various real - world scenarios.

#### 3.6.2 The protein and nucleic acid leakage

Based on the antimicrobial effectiveness of composite films, it was found that PLA/PVA-BDCA exhibited the best antimicrobial properties. This section further investigates the leakage of intracellular proteins and nucleic acids from two bacterial strains treated with the PLA/PVA-BDCA composite film, using untreated bacteria as the control group. The results are shown on Fig. 4(D, E). Firstly, the protein leakage was analyzed using A260 values to estimate the protein content. The results indicate that, compared to the control group, there was a significant increase in A260 values for both bacterial strains under both light and dark conditions. This suggests that the antimicrobial film treatment caused substantial leakage of intracellular proteins from the bacteria. Next, the nucleic acid leakage was examined using A280 values to estimate the nucleic acid content. The results show a significant increase in A280 values for both bacterial strains compared to the control group, under both light and dark conditions. This indicates that the composite film treatment also caused significant leakage of nucleic acids from the bacteria (W. Li et al., 2024).

Combining these observations with existing antimicrobial mechanisms (Jiang et al., 2024), it can be inferred that the antimicrobial composite film likely disrupts the bacterial cell membrane structure, leading to the leakage of intracellular proteins and nucleic acids (Qin et al., 2024; Y. Zhang et al., 2024). Given that the antimicrobial film shows no difference in effectiveness between Gram-negative and Gram-positive bacteria, it is hypothesized that the film generates a substantial amount of ROS, which damage the bacterial cell membrane (X. Guo et al., 2024; R. Zhang et al., 2024).

#### 3.6.3 The rechargeable mechanism

As shown in Fig. 4(A, B, C, D), the PLA/PVA-BDCA antimicrobial composite film exhibits significant antimicrobial effects against two bacterial strains during five cycles of use. To further investigate why the antimicrobial film can be reused five times, this study tested the generation of free radicals by the PLA/PVA-BDCA antimicrobial composite film during its reuse process, with results presented in Fig. 4(E, F). The composite membrane could generate ROS after each illumination, and there is no significant difference between different times (p ˃ 0.05). The findings indicate that during each use, a substantial number of free radicals is produced, meaning that each time the antimicrobial effect diminishes, new free radicals can be generated through UV regeneration. Furthermore, the regeneration of the antibacterial ability of the composite film is achieved through the light regeneration of ROS (Si et al., 2018).

Combining these results with related research, this study hypothesizes the mechanism of antimicrobial performance regeneration, as illustrated in Fig. 4(F). Under the combined action of UV light, hydrogen protons, and oxygen, free radicals can be cyclically generated, which corresponds with the experimental results of this study. Concurrently, other researchers have also developed similar reusable antimicrobial films, with the primary mechanism being the cyclical generation of free radicals, though the methods of generating these free radicals differ (Yang et al., 2023). The research results of Wu et al. (2024) show that the regeneration of the antibacterial performance of their composite film requires the use of chemical solutions. Compared to other studies, the method of UV light regeneration used in this research is simpler and more advantageous (N. Zhang et al., 2024).

### 3.7 Packaging effect

The selection of fresh cucumbers as the testing matrix is based on their relevance as a representative model for perishable agricultural products. Cucumbers are highly prone to microbial spoilage, making them ideal for evaluating the practical application of antimicrobial films. This choice aligns with the study's goal to demonstrate the film's efficacy in real-world preservation scenarios.

**Fig. 5:**
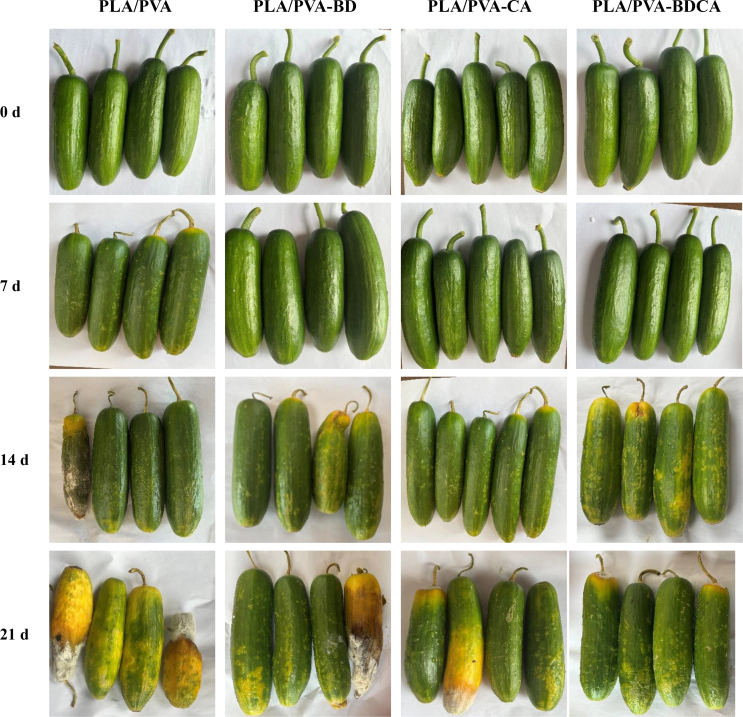
Photos of cucumber fruits during storage (0–21 d).

The cucumbers in the top row, representing day zero, are fresh with vibrant green coloration and no visible microbial growth or spoilage. Over the 21-day storage period, differences in appearance due to packaging become increasingly apparent across the rows (Fig. 5). By comparing each row, the progression of spoilage and microbial activity is clear (Feng et al., 2024; Zhang et al., 2024). In the second row, cucumbers maintain a relatively fresh appearance, with only slight discoloration and minimal surface deterioration. This indicates that PLA/PVA-BD, PLA/PVA-CA, and PLA/PVA-BDCA are effective at preserving freshness for approximately 7 days. The cucumbers in this row display no visible signs of mold or advanced spoilage, suggesting that the chosen packaging types might inhibit microbial growth to some extent within the first week of storage. In the third and fourth rows, corresponding to approximately 14 days of storage, more distinct differences emerge across the various packaging treatments. PLA/PVA begin to show yellowing, which suggests enzymatic degradation and chlorophyll breakdown-a common sign of aging in cucumbers. Additionally, slight signs of microbial activity, such as small patches of mold, can be observed on PLA/PVA. The final row, representing 21 days of storage, demonstrates the most significant deterioration. Cucumbers show considerable yellowing and darkening, with extensive microbial colonization visible in PLA/PVA, PLA/PVA-BD, and PLA/PVA-CA. Overall, these results illustrate that PLA/PVA-BDCA plays a critical role in maintaining cucumber freshness, particularly in controlling microbial growth and delaying spoilage.

In conclusion, this study visually demonstrates the influence of packaging on the preservation of cucumbers over a 21-day storage period. The presence of microbial growth and spoilage symptoms, such as yellowing and mold, underscores the importance of selecting suitable packaging materials and conditions to extend the postharvest life of cucumbers (Bakar et al., 2024). Further investigations could focus on optimizing packaging conditions, such as oxygen and carbon dioxide levels, to inhibit microbial proliferation and minimize spoilage, thus enhancing the commercial viability and consumer acceptance of cucumbers during extended storage.

## 4 Conclusion and prospects

In this study, electrospinning technology was utilized to in-situ composite a PVA film layer onto the PLA. Based on the molecular grafting principle, a photosensitive initiator, BD, which exhibits antimicrobial properties in solution, was immobilized on the surface of the PVA film layer. Subsequently, CA was attached to the BD molecule to prepare the composite film. SEM results revealed that the surface molecular grafting treatment had no significant impact on the morphology of the film surface. The nanofiber structure on the film surface remained intact. Nuclear magnetic resonance (NMR) spectroscopy indicated that BD, CA, and BDCA were chemically bonded to PVA rather than physically absorbed, thus confirming the successful molecular grafting on the surface of the composite film. The surface contact angles of the composite films all increased. Among them, the PLA/PVA-BDCA composite film showed the largest increase rate, reaching 44 %, which indicated an enhancement in the hydrophobicity of the composite film. Irrespective of light or dark conditions, the composite film demonstrated antibacterial effects against *Escherichia coli* and *Listeria monocytogenes*. The antibacterial rate under light conditions was approximately twice that under dark conditions. The PLA/PVA-BDCA composite film exhibited the highest antibacterial rate in both light and dark conditions, with the total colony count decreasing by about 3 log units. The PLA/PVA-BDCA composite film generated the highest amount of free radicals, reaching around 400 μg/g, which was more than twice that of the other two groups. After UV irradiation, the antibacterial performance and free-radical-generating capacity of the PLA/PVA-BDCA composite film could cycle five times. This indicated that the regeneration of the antibacterial function was achieved through the cyclic generation of free radicals under UV light. In the PLA/PVA-BDCA treatment group, no significant microbial growth was observed on the surface of cucumbers during the 21-day storage period.

This project focused on developing a composite membrane with renewable antibacterial functionality via light exposure for food preservation. Key advantages included its excellent efficacy against Gram-negative/positive model bacteria, with antibacterial function renewable for up to five cycles under light, enhancing reusability. The ROS-based mechanism ensured broad-spectrum activity against pathogens, while reducing chemical preservative use aligned with industry “clean label” trends. Limitations comprised reliance on a single cucumber variety and two strains, limiting generalizability to other produce/microbes. Lack of comparative data with traditional methods hindered assessment of competitive edge. Missing biodegradability testing and long-term logistics data also constrained sustainability and commercialization evaluations. Future research should optimize formulations, expand application scope, and address environmental compatibility to enhance practical value.

## CRediT authorship contribution statement

**Mi Li:** Writing – review & editing, Writing – original draft, Software, Methodology, Formal analysis. **Yunge Zhang:** Writing – review & editing, Methodology. **Quanlin Li:** Software. **Weirong Yao:** Writing – review & editing, Investigation, Funding acquisition. **Jian Ju:** Writing – review & editing, Software. **Yanli Ma:** Writing – review & editing, Funding acquisition, Formal analysis.

## Declaration of generative AI and AI-assisted technologies in the writing process

During the preparation of this work, the author(s) used Doubao, an AI language-processing tool, in order to improve the language readability of the manuscript. This tool was mainly utilized to refine sentence structures, correct grammar and spelling mistakes, and enhance the overall flow of the text. After using this tool/service, the author(s) reviewed and edited the content meticulously as needed. We carefully examined every section to ensure that the information presented was accurate, consistent with our research findings, and adhered to the academic integrity standards. The author(s) take full responsibility for the content of the publication, including all data, interpretations, and conclusions.

## Declaration of competing interest

The authors declare that they have no known competing financial interests or personal relationships that could have appeared to influence the work reported in this paper.

## Data Availability

The data that has been used is confidential.
